# An up-to-date review of decursin and its anti-cancer activities

**DOI:** 10.17179/excli2025-8999

**Published:** 2025-11-07

**Authors:** Chang Ha Park

**Affiliations:** 1 Department of Smart Farm, Namseoul University, 91 Daehak-ro, Seonghwan-eup, Seobuk-gu, Cheonan-si, Chungcheongnam-do 31020, Republic of Korea

## ⁯⁯⁯

Decursin (a pyranocoumarin compound) is a coumarin derivative found in *Angelica gigas*, particularly in its roots. The biosynthesis of decursin is primarily routed through the phenylpropanoid pathway, which is also responsible for the generation of several aromatic amino acids. Phenylalanine undergoes deamination by phenylalanine ammonia-lyase to produce cinnamic acid, which is subsequently hydroxylated at the para-position by cinnamate 4-hydroxylase to yield *p*-coumaric acid. Additional hydroxylation steps convert this intermediate into 2,4-dihydroxycinnamic acid, a key precursor for the formation of the coumarin skeleton and, ultimately, umbelliferone. The attachment of a 2-methylbut-2-ene side chain results in the formation of 7-demethylsuberosin, which then undergoes cyclization to generate decursinol. Subsequent modifications of decursinol give rise to its isomeric derivatives, decursin and decursinol angelate (Sestito et al., 2024[15]).

Decursin has gained significant attention as a potent antitumor agent owing to its substantial inhibitory effects on cancer progression and low toxicity in normal tissues (Hayashi et al., 2023[6]). Moreover, decursin exhibits anti-cancer effects across a wide spectrum of malignancies, including pancreatic, cervical, gastric, colorectal, prostate, liver, esophageal, head and neck, brain (glioblastoma), ovarian, non-small-cell lung, and inflammation-associated colorectal and skin cancers, B-cell lymphoma, FLT3-ITD acute myeloid leukemia, and hypoxia-driven tumors, underscoring its broad potential as a multi-targeted therapeutic agent. This review synthesizes recent advances in decursin research, with a particular focus on its anti-cancer mechanisms and therapeutic properties (Table 1(Tab. 1); References in Table 1: Ahmed et al., 2020[1]; Bhat et al., 2023[2]; Choi et al., 2016[3]; Fang et al., 2025[4]; Ge et al., 2020[5]; Joo et al., 2022[7]; Kim et al., 2016[10]; Kim et al., 2018[9]; Kim et al., 2021[11]; Kim et al., 2024[8]; Kweon et al., 2020[12]; Li et al., 2018[13]; Oh et al., 2019[14]; Yang et al., 2023[16]; Zhang et al., 2025[17]; Zhu et al., 2021[18]).

## Declaration

### Acknowledgments

Funding for this paper was provided by Namseoul University year 2025.

### Conflicts of interest

The author declares no conflict of interest.

### Artificial Intelligence (AI) - Assisted Technology

The author did not use artificial intelligence-based technologies.

## Figures and Tables

**Table 1:**
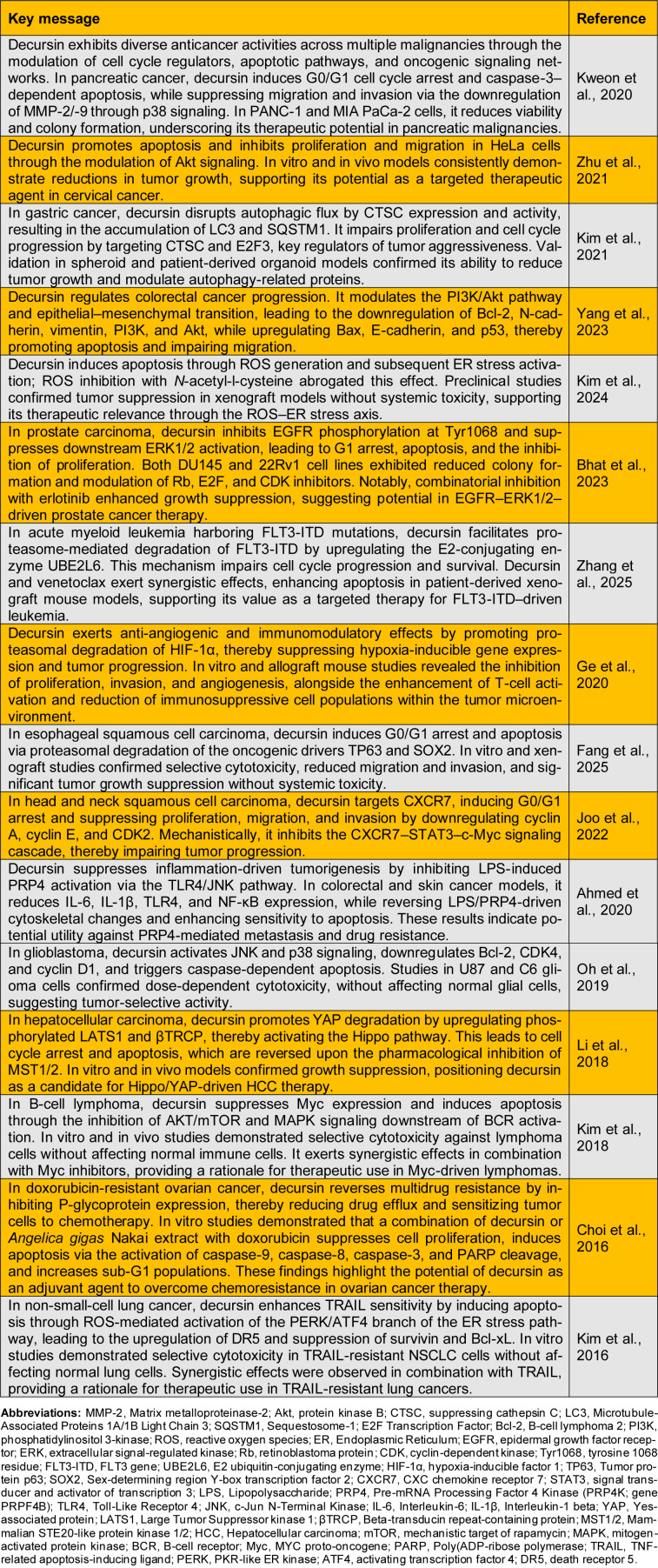
Overview of recent studies on the biological and pharmacological activities of decursin
